# Nutritional status is linked to muscle strength and perceived function in adults with muscular dystrophy: evidence for targeted nutritional interventions

**DOI:** 10.1017/S0007114525106119

**Published:** 2026-04-28

**Authors:** Meg Leaver, Christopher I. Morse, Paul Orme, Orla Flannery, Petra Kolic, Nathan Hodson

**Affiliations:** 1 Department of Sport and Exercise Sciences, Institute of Sport, Manchester Metropolitan University, Manchester, UK; 2 Neuromuscular Centre, Winsford, Cheshire, UK

**Keywords:** Muscular dystrophy, Nutrition, Strength and function, Quality of life

## Abstract

Muscular dystrophy (MD) encompasses inherited myopathies characterised by progressive skeletal and cardiac muscle degeneration, chronic inflammation and metabolic dysfunction. While emerging therapies show pre-clinical promise, few reach clinical translation, highlighting the need for supportive interventions to improve function and quality of life (QoL). Nutritional strategies may offer such benefits; however, limited data exist characterising diet in MD or associations with functional outcomes. This study assessed diet, nutritional status and associations with muscle strength, function and QoL in MD adults. Adults with MD (*n* 39; FSHD = 8, LGMD = 9 and Other = 22) and matched Controls (*n* 17) completed two 3-d food records, strength/function assessments and QoL questionnaires. Between-group differences were analysed using *t* tests or Mann–Whitney *U* and associations using Pearson’s r or Spearman’s Rho (*P* < 0·05). Compared with controls, individuals with MD consumed more energy (89 % *v*. 35 % exceeded RDI, *P* = 0·023), but less carbohydrate (–21 %, *P* = 0·013), sugar (–31 %, *P* = 0·004), protein (–15 %), BCAA (–31 %, *P* = 0·049) and vitamin C (–43 %, *P* = 0·009). MD participants demonstrated reduced muscle thickness, strength, function and reported lower QoL and physical capacity (all *P* < 0·05). Protein intake positively correlated with strength and function (*P* < 0·05); branched-chain amino acids intake was associated with lean mass (*r* = 0·442, *P* = 0·02) and strength (*r* = 0·372, *P* = 0·036). Findings indicate adults with MD consume excess energy but insufficient protein and micronutrients, supporting the need for adult MD-specific dietary guidance to optimise musculoskeletal health and QoL.

Muscular dystrophy (MD) is a heterogenous group of inherited myogenic disorders characterised by progressive atrophy, with severity and distribution varying by subtype. While these conditions broadly affect limb, axial and facial muscles, specific forms of MD extend to cardiac, pharyngeal, oesophageal and respiratory muscles^(1)^ particularly with disease progression. Specific forms of MD are classified based on the genetic mutation or pattern of muscle weakness^(2)^, however, can also be characterised by skeletal^(3)^ and cardiac muscle myopathy^(4)^, chronic inflammation^(5)^, disrupted signalling pathways^(6)^ and aberrant metabolism^(7)^. The clinical spectrum ranges from Duchenne MD, a non-ambulatory and life-limiting form, to the more ambulatory facioscapulohumeral dystrophy (FSHD) and limb-girdle MD (LGMD), which typically do not significantly limit lifespan, although cardiac or respiratory complications may occur to the heterogeneity of these conditions. With no curative treatments available, symptom management remains the primary approach to preserving strength, function and quality of life (QoL) in affected individuals. Given the role of nutrition in modulation of other chronic diseases^(8–10)^, there is growing interest in understanding the influence on clinical management in adults with MD.

Nutritional challenges in adults with MD are multifactorial, with facial weakness, tongue myotonia, oropharyngeal dysphagia, gastrointestinal dysmotility, distal muscle weakness and cognitive impairment collectively impairing food preparation and intake^(11,12)^. These issues are compounded by mobility limitations, socio-economic barriers and food accessibility, exacerbating nutritional inadequacies^(13,14)^. This is concerning given the link between low calcium, vitamin D and protein and reduced muscle health, alongside higher morbidity and mortality in other chronic conditions^(8,15,16)^. Evidence suggests age-related declines in lean mass are influenced by protein intake^(8)^, while insufficient calcium and vitamin D intakes are associated with fall risk^(17)^ contributing to diminished QoL in older adults^(18)^. Adequate intake of these nutrients represents a modifiable factor to support muscle strength, functional capacity and QoL in adults with MD. Previous research underscores the need to address modifiable lifestyle factors in MD, with diminished QoL linked to higher BMI and reduced capacity for daily activities^(19)^. While physical activity is beneficial, its limited by disease-specific constraints^(20)^, and therefore, dietary and nutritional interventions could offer a more practical, accessible strategy to enhance QoL in adults with MD.

Current evidence in dietary habits in MD^(1,21–23)^ is insufficient to ascertain if adults living with MD exhibit poorer nutritional intake compared with the general population or require condition-specific dietary recommendations. Studies by Motlagh *et al.* ^(1)^ and Amzali *et al.* ^(21)^ reported adults with MD (primarily myotonic dystrophy type 1and FSHD) exhibit low energy and inadequate micronutrients intakes; however, these investigations lack comparison to a non-dystrophic, healthy Control group, limiting interpretation of MD-specific effects. Although, micronutrient supplementation has been shown to have a positive impact on quadricep muscle strength in FSHD^(24)^, links with other key functional and QoL outcomes remain largely unexamined across the adult MD spectrum.

In MD, skeletal muscle contractile protein loss is driven by an imbalance between protein degradation and synthesis, resulting in negative protein balance^(25–27)^. Protein intake has a crucial role in protein metabolism^(28,29)^, directly stimulating muscle protein anabolism whilst indirectly suppressing catabolism via insulinogenic amino acids. Despite its importance, little is known about protein intake in adults with MD. Existing data, in Duchenne MD boys, indicate inadequate protein intake suggesting increased intake benefits muscle function^(23,30,31)^. Similarly, in adults with myotonic dystrophy type^(1)^, FSHD^(21)^ and LGMD^(7)^ insufficient dietary protein has been observed; however, associations with functional outcomes or QoL remain unexplored.

Metabolic comorbidities, including increased insulin sensitivity^(32,33)^ and vitamin D deficiency, are prevalent in MD^(34,35)^. Given the established roles of insulin sensitivity^(36,37)^ and vitamin D in skeletal muscle function^(38,39)^, these factors act synergistically with protein intake influencing disease progression and patient outcomes. Identifying habitual diet in MD could inform the development of condition-specific dietary guidelines, which are absent. Such insights would provide a basis for targeted, interventions aimed at improving muscle function, metabolic health and QoL in this population.

This study, therefore, aimed to evaluate dietary intake, quality of life, muscle morphology and physical function in adults with MD relative to healthy controls and explored diet-related associations in the MD cohort.

## Materials and methods

### Participants

In total, 104 adults with MD receiving physiotherapy treatment at The Neuromuscular Centre (Winsford, Cheshire, UK) were recruited. MD participants were eligible to participate if they had received a diagnosis of MD (any condition on the MD spectrum as defined by Muscular Dystrophy UK)^(40)^ were aged between 18 and 80 years (chronological age), had any level of functional ability and were regularly attending the centre for physiotherapy, defined as minimum 1 visit per month. Participants were excluded if they had a gastronomy feeding tube, additional chronic health conditions unrelated to their MD diagnosis or cognitive processing difficulties. Those with gastrostomy tubes were excluded as they lacked autonomy over food choices and were under dietetic care, with nutrition therefore controlled and optimised. Potential participants were identified by physiotherapists and subsequently approached for possible recruitment by the first author. Controls unrelated to the MD participants and free of any long-term health conditions including cardiac, respiratory, metabolic or neurological condition and aged 18–80 years were also invited to participate. Informed consent was obtained from all participants prior to study enrolment. Data collection complied with the principles of the Declaration of Helsinki, and the study was approved by the Manchester Metropolitan University Institute of Sport Research Ethics Committee (project ID: 48048).

### Procedure

This project was conducted in three parts: (1) dietary assessment (2) physiological outcome measurement and (3) questionnaires. Participants completed two food records and questionnaire batteries separated by ∼8 weeks, with a testing session conducted at an intermediate point to assess physiological outcome measures. The same equipment was used for all participants, except for seated scales, which were employed to measure body mass in non-ambulatory MD participants. Ambulatory status was defined by the ability to complete 10 m walk and timed-up and go tests with optional walking aids. Questionnaires assessing physical activity, perceived function and quality of life were completed alongside the first food record, followed by nutrition knowledge questionnaires with the second food record. These were administered online or via paper, with support provided by the lead investigators at participants’ request.

### Dietary assessment

Participants were asked to keep a weighted record of their habitual diet over a 3-d period (2 weekdays and 1 weekend day) and told to record intakes immediately after consumption along with preparation method and brand names to ensure accuracy. Participants could complete this on written hard copies or a smart phone app (Libra, Nutritics). Food records were repeated after ∼8 weeks to obtain a more accurate representation of habitual dietary patterns^(41,42)^, with the average of both records used for analysis by a nutritionist (Nutritics). Level of ultra-processed food intake was assessed using the NOVA ultra-processed food classification system^(43)^, and overall diet quality was evaluated with the Healthy Eating Index (HEI-2020)^(44)^, these subjective assessment tools were applied independently by separate investigators, yielding a 98 % inter-rater agreement. Comparisons between groups were made in accordance with sex- and age-specific dietary and physical activity recommendations from the UK government^(45)^.

### Physiological outcomes

#### Anthropometry

Height was calculated in all participants as point-to-point of arm span (index finger, elbow, shoulder and across the midline) to reflect the method used on all non-ambulatory participants^(46–48)^. To account for the known discrepancy between height and arm span measures, a correction was applied to the raw data^(49,50)^. Body mass in MD participants was measured by a digital seated scales system (6875, Detecto), with weight of shoes, slings and splints deducted from gross weight. In the Control group, mass and stature were measured using a digital scale (Seca model 873) and a wall mounted stadiometer (Harpenden stadiometer, Holtain Crymych). Bioelectrical impedance analysis (Bodystat 1500, Bodystat Ltd.) was utilised for assessments of body composition and completed in a minimum 2-hour fasted state.

#### Muscle thickness assessment

All muscle thickness (MT) measures were completed with the same B-mode ultrasound system (MyLab Gamma, Esaote) and took place with the participant seated with hip and knee maintained at 90°. MT was assessed at both the lateral and medial aspects of the dominant forearm: 30 % of the proximal distance between the styloid process and the distal insertion of the biceps brachii muscle into the radial tuberosity. During measurement, the participant was seated with the dominant arm resting on a standardised table, the shoulder flexed to 30–45°, the elbow at 135°, mid pronation/supination, 15–30° wrist extension and 0–15° ulnar deviation. To standardise positioning, a tennis ball was gently held to minimise voluntary and involuntary forearm muscle contractions. Two MT measurements were obtained: MT-ulna and MT-radius, defined as the perpendicular distance between the subcutaneous adipose tissue–muscle interface and the muscle–bone interface of the ulna and radius, respectively. An integrated electronic calliper on the ultrasound (MyLab Gamma, Esaote) was used to measure the distance between the two interfaces, with measurements conducted in triplicate and a mean value obtained. The average coefficient of variation in MT-radial and MT-ulna measures was 2·95 % and 2·52 %, respectively. This method has previously been used in the assessment of sarcopenia in geriatric populations^(51)^ and assessing muscle thickness in a young healthy population^(52)^, showing strong intra-rater reliability.

#### Muscle strength

All maximum voluntary contraction measures were conducted with participants seated, maintaining hip and knee at 90°. Shoulder abduction (AA) and elbow flexion maximum voluntary contraction were assessed with a hand-held dynamometer (Lafayette Instrument), whilst hand grip strength was assessed with a JAMAR handheld dynamometer (Smith and Nephew, NY, USA). Maximal contractions were held for ∼5s with strong verbal encouragement, and participants completed three trials for each movement separated by a 2-minute rest interval and the highest value was used. If the standard position was unachievable due to physical limitations in MD, alternative positions were adopted (e.g. resting the arm by the side or on the lap).

#### Functional tasks

All ambulant participants completed two timed functional tests: 10 m walk and timed up and go, each commonly used in neuromuscular conditions and well validated^(53–55)^.

The timed-up and go test involves rising from a standard armchair, walking 3 meters, performing a 180° turn, returning and sitting down. Walking aids were permitted if needed, and time was recorded by the principal investigator.

#### Blood samples

Capillary blood samples were obtained from the fingertip (∼0·5 ml) using lancets (Accu-Check Safe-T-Pro). The VHC Vitamin D Quantitative Vitamin D Test (Vitality Health Check) was used to determine 25OH vitamin D levels in participants. This point of care system has a detection range from 3 ng/ml (7·5 nmol/l) to 100 ng/ml (250 nmol/I) and has good agreement with mass spectrometry-derived 25OH vitamin D concentrations (*r* ^2^ = 0·90, manufacturer testing)^(56)^.

Glycated haemoglobin (HbA1c) levels were measured using Quo-Test HBA1c system (QuoTest) according to manufacturer’s instructions.

### Questionnaires

#### Physical activity

The Physical Activity Scale for Individuals with Physical Disabilities assesses physical activity in epidemiologic studies involving individuals with disabilities. Adapted from the validated Physical Activity Scale for the Elderly^(57–59)^, Physical Activity Scale for Individuals with Physical Disabilities requires participants to recall their activity frequency over the past seven days and estimate daily duration. The Physical Activity Scale for Individuals with Physical Disabilities score is calculated by multiplying daily hours per activity by corresponding MET values and summing across items.

Activities of daily living was assessed by utilising the twenty-two-item Nottingham Extended Activities of daily living Scale, previously validated in various clinical conditions^(60–62)^. Respondents record completed activities over the past few weeks, with possible answers of ‘On your own’, ‘On your own with difficulty’, ‘With help’ or ‘Not at all’. For enhanced sensitivity, scores were assigned using a Likert scale of ‘0–1–2–3’, for these answers. Therefore, total scores ranged from 0 to 66, with higher scores indicating greater independence^(63,64)^.

#### Perceived function

Perceived function was assessed using the Bathel Index, ABILHAND+ and Lower Extremity Functional Scale (LEFS) questionnaires, each having high reliability and validity in neuromuscular/clinical populations^(65–70)^. The Bathel Index is a tenitem scale assessing functional independence in mobility and personal care. Each item is scored 0 (unable), intermediate score 5 (with assistance) or a maximum score 10 (independently) with total scores ranging from 0 to 100 and categorised as 0–20 (total dependence), 21–61 (severe dependence), 62–90 (moderate dependence), 91–99 (mild dependence) and 100 (complete independence).

The ABILHAND+ is a twenty-two-item questionnaire assessing perceived difficulty in performing manual tasks, adapted from the original ABILHAND using the Rasch model for patients with neuromuscular disorders^(71)^. The cumulative score is scaled along a unidimensional continuum, with higher logit values indicating greater perceived ability.

The LEFS assesses lower extremity function in patients with musculoskeletal conditions, with scores ranging from 0 (significant limitations) to 80 (no constraints)^(69)^.

#### Quality of life

Participants completed the SF-36v2 questionnaire, which assesses QoL across eight domains; General Health, Role-Functioning Physical, Role Functioning Emotional, Physical Functioning, Mental Health, Vitality and Bodily Pain^(72)^. All ratings are graded on a scale of 100, where higher values indicate better perceived health, physical function and lower pain. The SF-36v2 has been validated extensively in various populations^(73–75)^.

#### Pain and fatigue

The Checklist Individual Strength was used to assess subjective fatigue, assessing four-dimension fatigue across twenty items using a seven-point Likert scale and previously shown to have high reliability and discriminative validity^(76,77)^. Higher scores indicate increased fatigue, concentration issues, reduced motivation or lower activity. A cut-off score of 35 is used to identify severe fatigue and effectively differentiates between patient groups and healthy subjects^(78,79)^.

A digital Visual Analogue Scale was used to assess pain experienced by participants over the past 7 d. Visual Analogue Scale is a validated tool for pain measurement across various clinical conditions, including MD^(80,81)^. Participants marked a 10 cm line ranging from ‘No Pain’ to ‘Worst Possible Pain,’ with the distance from ‘No Pain’ measured to quantify pain intensity.

#### Nutrition knowledge

Nutrition knowledge was assessed using a revised version of the Nutritional Knowledge Questionnaire developed and validated in the UK^(82)^. The updated eighty-eight-item tool reflects current UK and international nutritional guidelines and has proven to be reliable, valid and sensitive to changes in knowledge^(83)^. See online Supplementary Questionnaire 1.

A Short Food Literacy Questionnaire was also completed, which assesses functional, interactive and critical food literacy skills. We adapted the validated Swiss Short Food Literacy Questionnaire to reflect UK references, such as the UK Eatwell plate instead of the Swiss Food Pyramid^(84)^. The instrument consists of twelve self-rated items, with responses on four- or five-point Likert scales (e.g. ‘very bad’ to ‘very good,’ ‘disagree strongly’ to ‘agree strongly’).

### Statistical analysis

All statistical analysis was conducted using SPSS (version 29 for MacOS; SPSS). Differences in nutritional intake, physical function, physiological outcomes and questionnaire response scores between two groups (MD *v*. Control) were analysed using independent samples *t* test or Mann–Whitney *U* tests. Depending on normality outcome measures, comparisons between multiple groups (FSHD, LGMD, Other and Control) were analysed using a one-way ANOVA, with Bonferroni-corrected *t* tests performed *post hoc* if a significant main effect was observed. Non-parametric data for these comparisons were analysed using Kruskal–Wallis tests followed by corrected Mann–Whitney U pairwise comparisons where appropriate main effects were observed. Associations between nutritional intake, physical outcome measures, perceived function and QoL were tested using Pearsons’s or Spearman’s rank correlation analysis for parametric and non-parametric data respectively. Significance for all statistical tests was set at *P* ≤ 0·05. Data are presented as means (standard deviation (sd)), unless stated otherwise.

## Results

**Figure 1.**
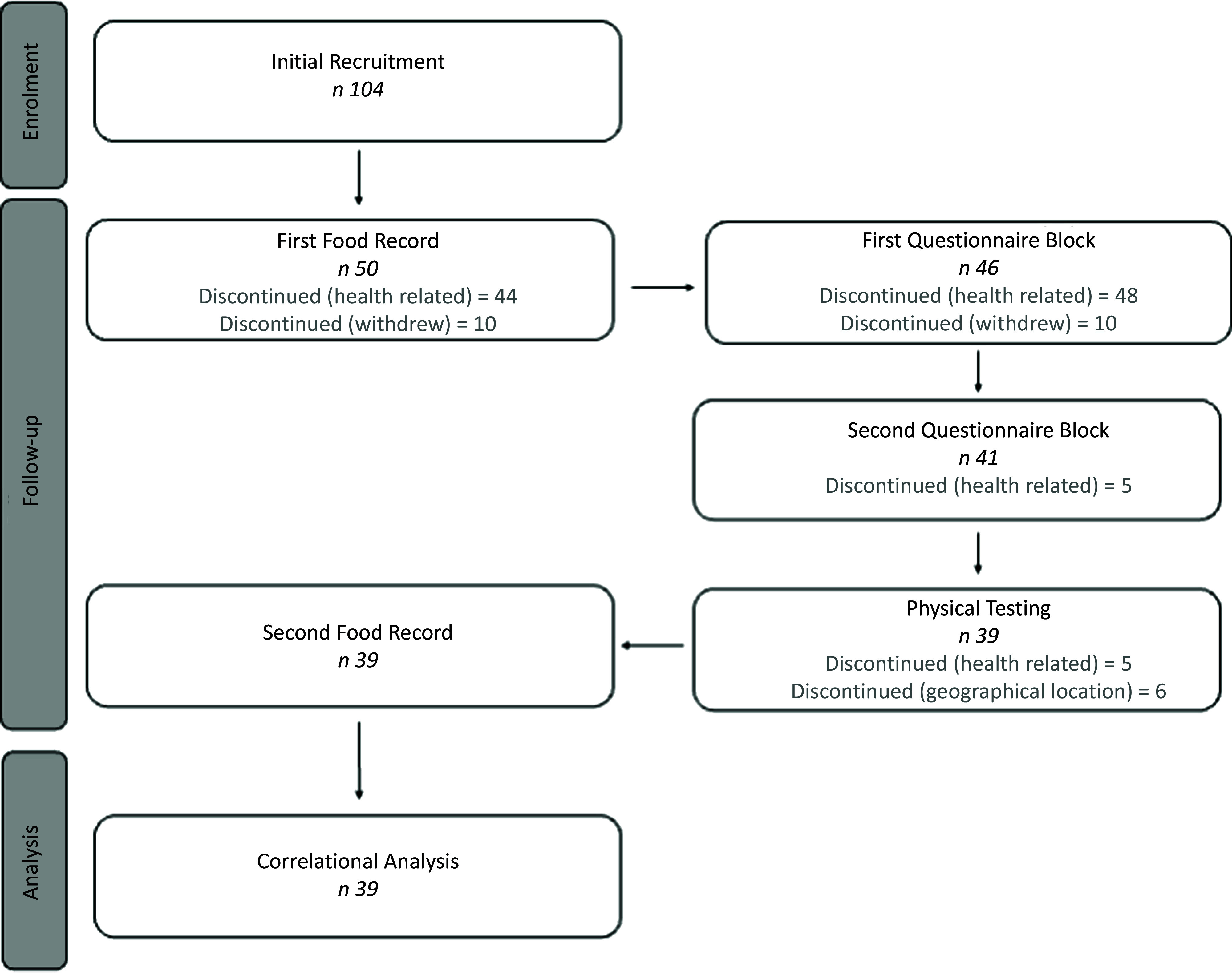
Diagram illustrating the enrolment, allocation, follow-up and analysis stages of the MD group. A total of 104 participants were recruited. During follow-up, participant discontinuations were recorded with reasons specified: 10 voluntary withdrawals in the first block. Additional losses occurred later due to health issues (e.g. General disease progression and gastronomy tube feeding) and geographical location issues. Ultimately, thirty-nine participants from the MD group and seventeen from the Control group were included in the final analysis. MD, muscular dystrophy.

Of the 104 MD participants initially recruited, thirty-nine completed the full protocol, while an additional two completed only the questionnaires (Figure 1). The high withdrawal rate and incomplete participation were primarily due to health-related factors, including disease progression, illness and the need for gastrostomy tube feeding. For the Control group, seventeen healthy controls were evaluated.

### Participant characteristics

Table 1 displays anthropometric data of thirty-nine participants with MD and seventeen healthy controls. The MD group (Table 2) consisted of participants having facioscapulohumeral muscular dystrophy (FSHD, *n* 8) and limb-girdle muscular dystrophy (LGMD, *n* 9) and ‘other’ conditions (those which had less than nine participants, *n* 22). ’Other’ conditions included Charcot-Marie-Tooth disease, myotonic dystrophy type 2, inclusion body myositis and spinal muscular atrophy types 2 and 3. All variables other than age were not significantly different between cohorts.

**Table 1.** Anthropometric characteristics of participants

	MD^†^		Control	
	(*n* 39, M = 22, F = 15)		(*n* 17, M = 7, F = 10)	
	Mean	sd	Mean	sd
Age (years)	61	14*	38	12
Height (m)	1·73	0·11	1·74	0·09
Weight (kg)	80·61	19·68	85·71	16·71
BMI^‡^ (kg/m^2^)	26·78	4·99	28·5	5·89
Lean mass (%)	64·53	8·34	67·38	10·29

MD, muscular dystrophy.

Values are means (sd). *MD significantly different from Control (*P* < 0·01).^†^MD, muscular dystrophy participants ^‡^BMI.

**Table 2.** Anthropometric characteristics of MD subdivided based on condition

	FSHD^†^		LGMD^‡^		Other	
	(M = 4, F = 4)		(M = 3, F = 6)		(M = 15, F = 7)	
	Mean	sd	Mean	sd	Mean	sd
Age (years)	61·5	15·74	60·22	14·2	59	14·4
Height (m)	1·72	0·11	1·67	0·08	1·76	0·12
Weight (kg)	74·73	11·59	71·7	14·94	85·62	21·50
BMI^§^ (kg/m^2^)	25·17	3·36	25·6	4·43	27·42	5·5
Lean mass (%)	64·53	9·57	65·74	11·78	69·22	10·53

Values are means (sd). ^†^FSHD, facioscapulohumeral muscular dystrophy; ^‡^LGMD, limb girdle muscular dystrophy; ^§^BMI.

### Dietary intake analysis

Energy and macronutrient intakes for MD and Control groups are presented in Table 3. Although the Control group consumed ∼369 kcal more than the MD group, overall energy intake did not differ significantly between groups. However, when expressed as a percentage of age-specific recommended daily intake (RDI), energy intake was greater in MD compared with Control (*P* = 0·023), with 89 % of the MD exceeding energy estimates compared with 35 % of the Control group (Table 3). Carbohydrate and total sugar intake were significantly higher in the Control group (*P* = 0·013 and *P* = 0·004, respectively), whereas no significant difference was observed in free sugar intake. No differences were observed in any other macronutrients.

**Table 3.** Diet assessment of macronutrient intake

	MD^†^				Control			
	(*n* 39)				(*n* 17)			
	Absolute		% RDI^‡^		Absolute		% RDI^‡^	
	Mean	sd	Mean	sd	Mean	sd	Mean	sd
Energy (kJ)	7267	1969			8821	2676		
Energy (kcal)	1733	470	139	48*	2102	638	114	32
Carbohydrate (g)	184·8	58·5*	62	18*	232·9	67·6	79	21
Sugars (g)	69·9	28·2*	234	94*	101·1	37·1	342	125
Free sugar (g)	23·1	17·0	77	57	25·8	20·1	86	67
Protein (g)	77·4	21·2	151	35*	93·4	31·2	187	56
Protein (g/kg/d)	0·98	0·27	131	37	1·11	0·35	149	46
Total fat (g)	71·7	21·2	135	63	85·0	31·0	113	35
Saturated fat (g)	26·7	9·9	117	59	29·8	13·4	104	38
Unsaturated fat (g)	45·0	12·5	192	82	55·1	21·8	184	79
Fibre (g)	19·0	5·8	64	19	24·5	11·4	82	38

MD, muscular dystrophy.

Values are means (sd). *MD significantly different from Control (*P* < 0·05). ^†^MD, muscular dystrophy participants; ^‡^RDI, recommended daily intake.

**Figure 2.**
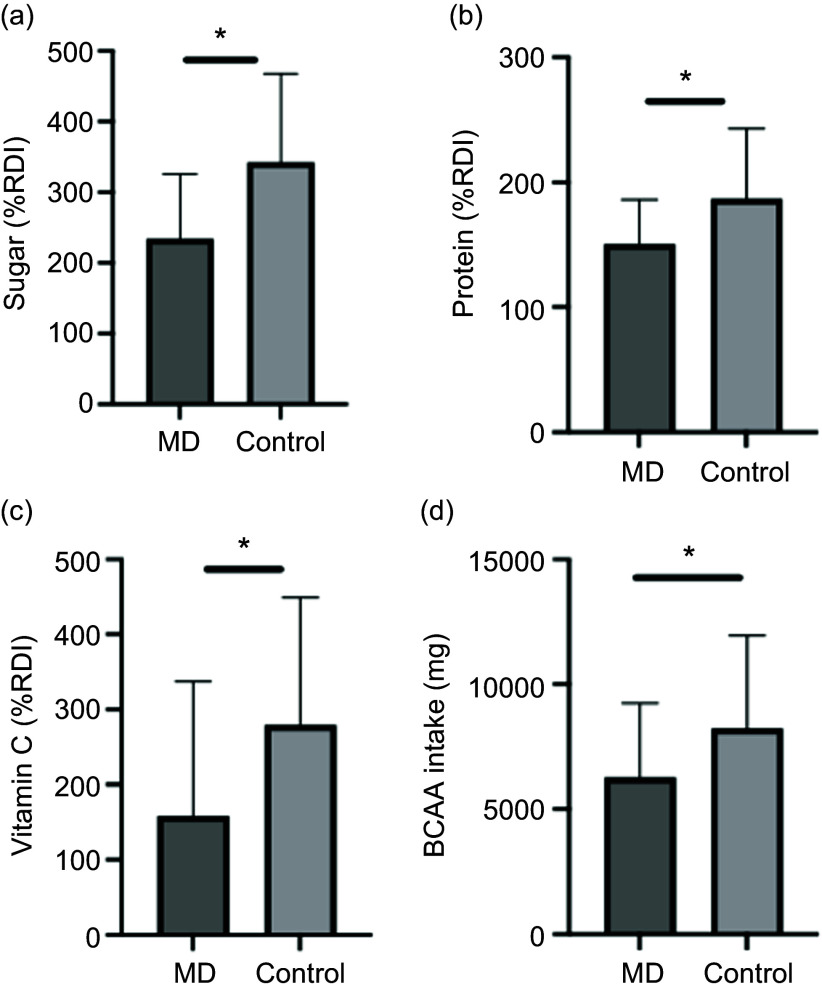
Effect of condition on nutrient intake as a percentage of RDI for sugar (a), protein (b) and vitamin C (c) and on BCAA intake (d). Data are shown as mean (sd). *MD significantly different from Control (*P* < 0·05). BCAA, branched-chain amino acids; MD, muscular dystrophy; RDI, recommended daily intake.

In Control, 24 % met the UK fibre recommendation (30 g/d), compared with 3 % in the MD group. Protein intake below the UK RDI (0·75 g/kg/d) was observed in 12 % of the Control group and 23 % of the MD group. Additionally, 56 % of the MD group exceeded the RDI for total fat intake, compared with 41 % in the Control group. Compared with the UK RDI, the Control group had 28 % higher intakes of sugar and 15 % higher protein intakes than the MD group (Figure 2(a) & (b)).

**Figure 3.**
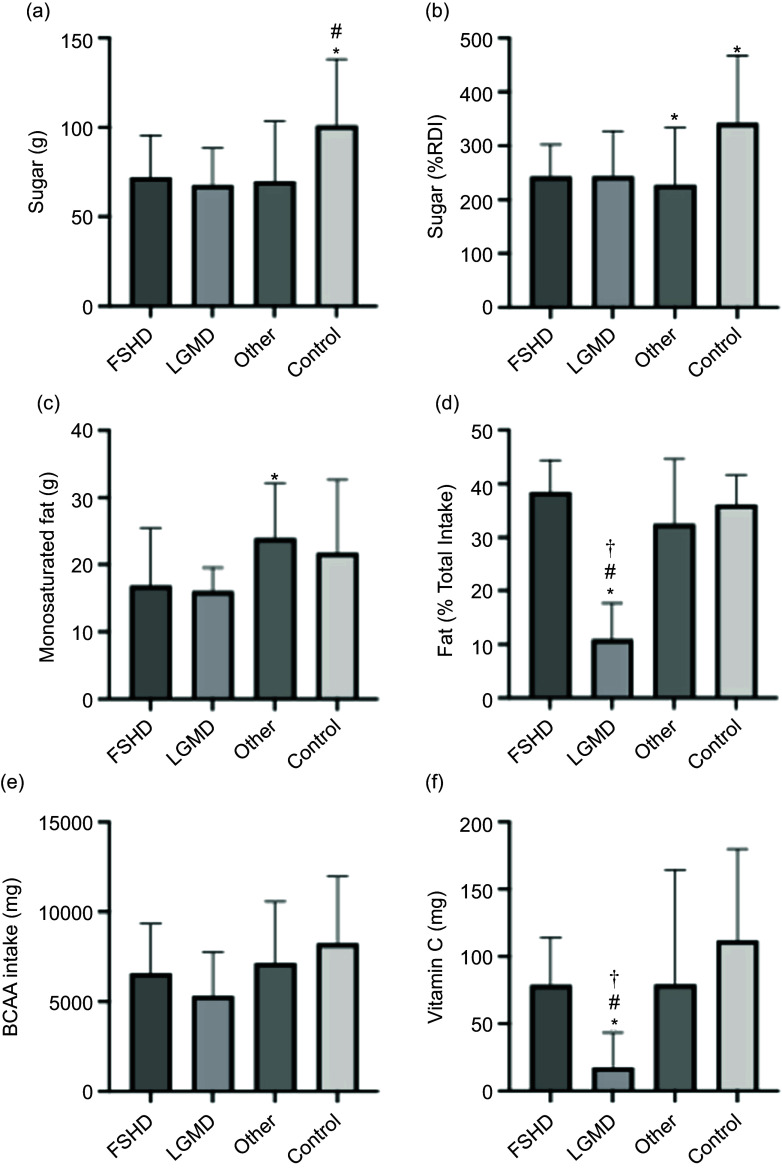
Nutrient intake comparisons among FSHD (*n* 8), LGMD (*n* 9), Other (*n* 22) and Control (*n* 17) groups. Data (mean (sd)) include (a) sugar (g), (b) sugar (%RDI), (c) monounsaturated fat (g), (d) fat (% total energy), (e) BCAA (mg) and (f) vitamin C (mg). Significant differences (*P* < 0·05) are indicated as follows: (a) *v. Other, # *v*. Control; (b, c) *v. Control; (d, e) *v. Other, # *v*. Control, ^†^ *v*. FSHD. BCAA, branched-chain amino acids; FSHD, facioscapulohumeral muscular dystrophy; LGMD, limb girdle muscular dystrophy; RDI, recommended daily intake.

When comparing macronutrient data across separate MD, both the LGMD and Other groups had significantly lower absolute sugar intake, 33 % and 31 %, respectively, than the Control group (Figure 3(a)). The LGMD group also had 25 % lower sugar intake relative to RDI compared with Controls (*P* = 0·05) (Figure 3(b)). Furthermore, the LGMD group (11 (sd 7) %) had a lower total fat intake as a percentage of total energy intake compared with all other groups (FSHD = 38 (sd 6), Other = 34 (sd 11), Control = 36 (sd 5), *P* = 0·003) (Figure 3(d)) and a lower monounsaturated fat intake (16·1 (sd 3·5) g) compared with Other (24 (sd 8·1) g, *P* = 0·048) (Figure 3(c)).

Although no differences were observed in overall NOVA ultra-processed food or HEI-2020 scores between groups (all *P* > 0·05) (data not presented), component analysis of the HEI-2020 indicated that the MD group consumed significantly less fruit than Controls (138 (sd 112) g *v*. 270·71 (sd 1) g; *P* = 0·012). The LGMD subgroup demonstrated the lowest fruit intake, 64 % lower than Controls and the lowest vegetable intake, 32 % lower than controls (online Supplementary Figure 1).

When comparing micronutrient intakes, a significant difference was only observed for vitamin C intake (*P* = 0·009, Table 4), with controls reporting 43 % higher intakes. However, on average, 71 % of the MD group and 66 % of the Control group did not meet the UK recommendations for all micronutrients listed in Table 4. When comparing micronutrient data across separate MD groups, only absolute vitamin C intake (mg) was different, with LGMD (17·34 (sd 25·94)) reporting lower intakes than in all other groups (FSHD = 79 (sd 34·9), Other = 79·3 (sd 84·8) and Control = 111·8 (sd 68), *P* < 0·05) (Figure 3(f)).

**Table 4.** Diet assessment of micronutrient intake

	MD^†^				Control			
	(*n* 39)				(*n* 17)			
	Absolute		% RDI^‡^		Absolute		% RDI^‡^	
	Mean	sd	Mean	sd	Mean	sd	Mean	sd
*n*-3 (g)	1·14	0·95	83	74	1·15	1·19	82	72
Cholesterol (mg)	235	148	79	50	241	163	85	51
Na (mg)	2135	638	89	27	2132	507	89	21
Ca (mg)	707	294	103	42	750	340	107	49
Potassium (mg)	2435	823	71	23	2643	914	74	27
Mg (mg)	224	76·8	79	25	278	101	92	26
Fe (mg)	8·91	3·18	92	41	9·28	3·56	83	45
Vitamin D (µg)	2·98	2·45	28	24	6·18	11·12	62	15
Vitamin B_12_ (µg)	6·13	10·77	261	213	4·03	2·6	269	173
Vitamin C (mg)	63·77	70·41*	154	181*	111·74	67·96	279	170

MD, muscular dystrophy.

Values are means (sd). *MD significantly different from Control (*P* = 0·009). ^†^MD, muscular dystrophy participants; ^‡^RDI, recommended daily intake.

In relation to specific amino acids subgroups, branched-chain amino acids intake was significantly higher in the Control group (8226 (sd 3723) mg) compared with the MD group (6275 (sd 2963) mg, *P* = 0·049) (Figure 2(d)); however, no difference was found when assessed as separated MD groups (Figure 3(e)).

### Skeletal muscle size, strength and function

Skeletal muscle size, strength and function findings are summarised in Table 5. Of the thirty-nine MD participants, only thirteen (FSHD = 4, LGMD = 2, Other = 7) completed the walking performance measures due to mobility limitations. All strength measures were significantly greater in the Control group compared with the MD group (all *P* < 0·05), with strength deficits in the MD group ranging from 42 % to 60 %. Additionally, the MD group exhibited slower performance across all walking assessments. Forearm radial muscle thickness was 26 % greater in controls (*P* = 0·013); however, no significant difference in ulna muscle thickness was observed between groups.

**Table 5.** Muscle thickness, function and strength measures

	MD^†^		Control	
	(*n* 39)		(*n* 17)	
	Mean	sd	Mean	sd
Grip strength (kg)	24·3	17·1*	48	15·7
Elbow flexor strength (kg)	7·7	6·2*	18·8	11·7
Arm abduction strength (kg)	9·2	6·9*	15·8	10·3
Radial muscle thickness (mm)	19·6	4·4*	23·2	5·4
Ulna muscle thickness (mm)	24	4·9	24·2	5·1
	(*n* 13)		(*n* 17)	
10 m walk (s)	11·4	3·7*	7·9	1·3
Timed up and go (s)	12·9	6·4*	7·3	0·6

MD, muscular dystrophy.

Values are means (sd). *MD significantly different from Control (*P* < 0·05). ^†^MD, muscular dystrophy participants; ^‡^HBA1c, glycated Hb.

**Figure 4.**
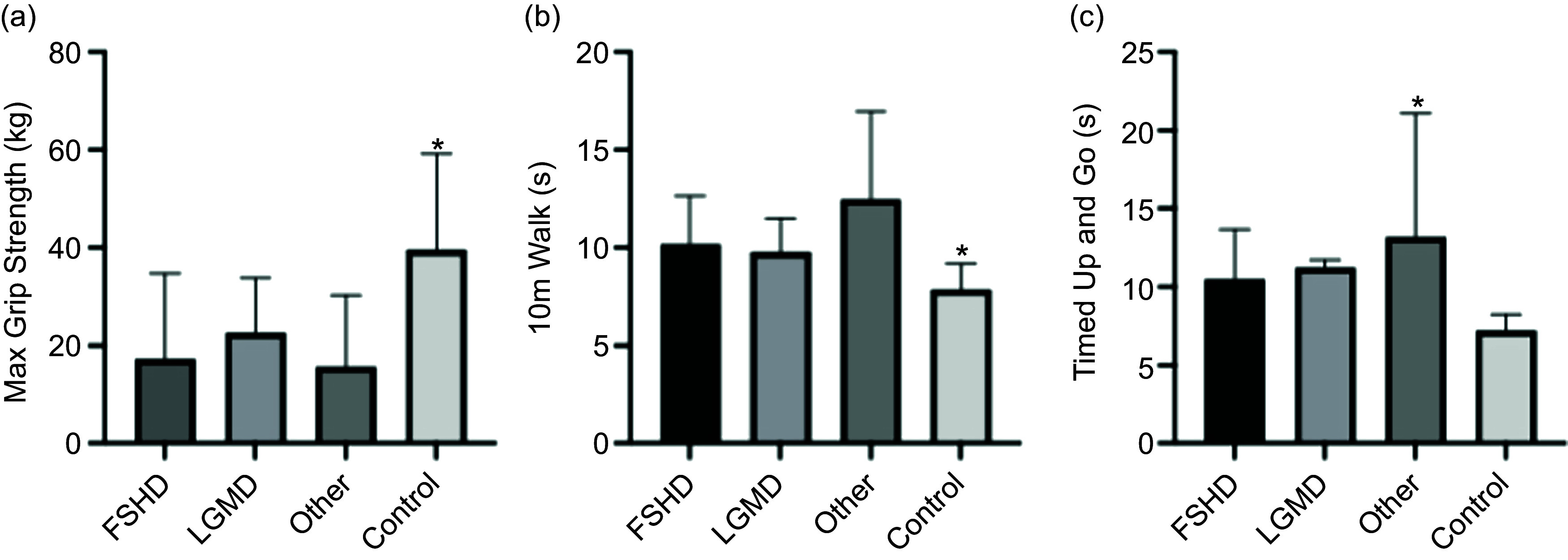
Grip strength measures in FSHD (*n* 8), LGMD(*n* 9), Other (*n* 22) and Control groups (*n* 17) and walking performance (10 m Walk, TUG) (FSHD = 4, LGMD = 2, Other = 7 and Control = 17). Values are mean (sd) *indicates difference *v*. Other (*P* < 0·05). FSHD, facioscapulohumeral muscular dystrophy; LGMD, limb girdle muscular dystrophy; TUG, timed-up and go.

1Analysis of separate MD groups displayed 60 % lower maximum grip strength for the other group compared with Control (*P* = 0·005, Figure 4(a)). Other also recorded a 37 % slower time in 10 m and a 44 % slower time in timed-up and go compared with Control (*P* < 0·02) (Figure 4(b), (c)). No other separated group differences were reported. Age was not significantly correlated with any of the muscle or functional outcome measures. Accordingly, age was not identified as a confounding factor in any muscle or strength analyses.

### Quality of life, perceived function and nutrition knowledge

**Figure 5.**
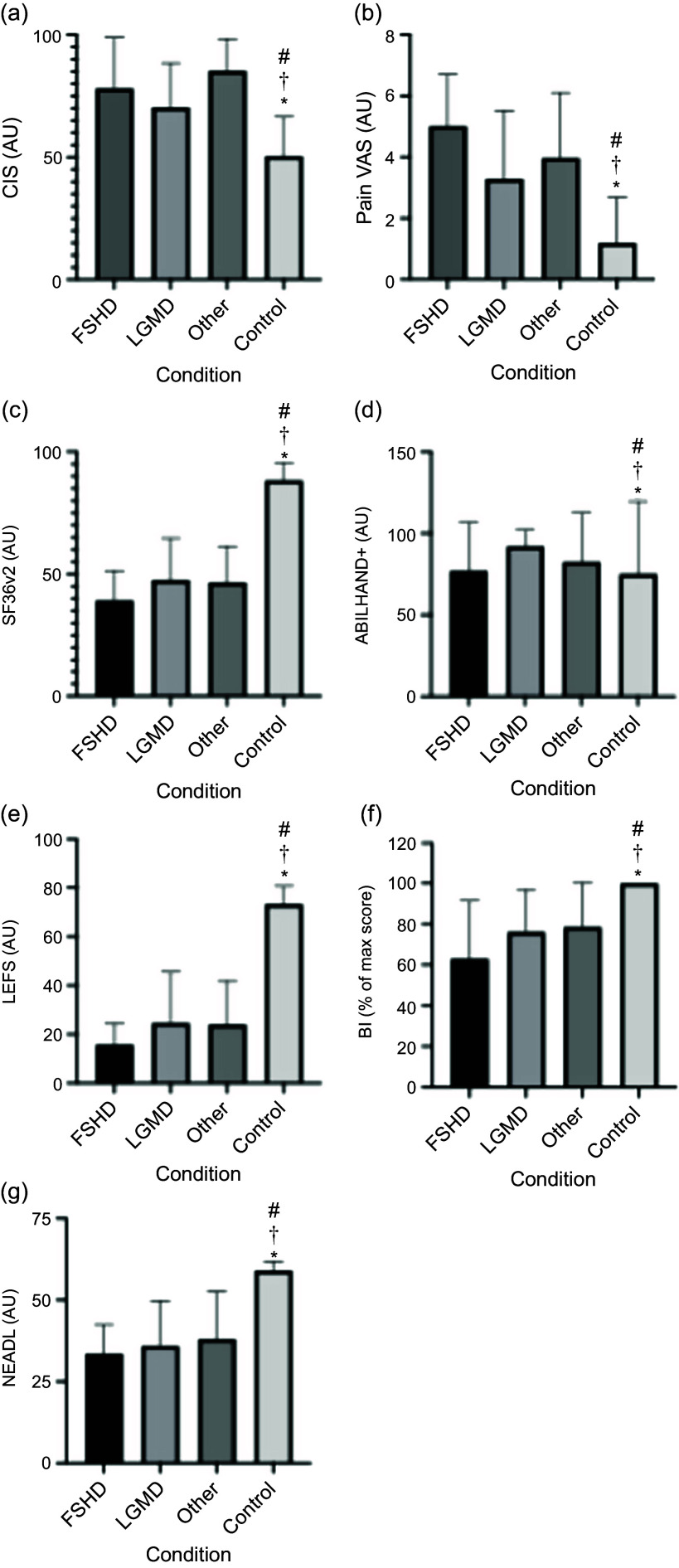
QoL and perceived functional ability across multiple assessment tools. (a) Checklist Individual Strength (CIS), where a higher score indicates increased fatigue. (c) SF-36v2, (d) ABILHAND+ (scored as a percentage of the maximum score), (e) Lower Extremity Functional Scale (LEFS), (f) Barthel Index (BI, scored as a percentage of the maximum score) and (g) Nottingham Extended Activities of Daily Living (NEADL), where higher scores reflect worse functioning or reduced QoL. (b) Pain Visual Analogues scale (Pain Visual Analogue Scale) A higher score denotes increased pain or fatigue. Data are presented as mean (sd). Significant differences (*P* < 0·05) are indicated as follows: *v. Other, #*v*. Control, ^†^ *v*. FSHD. BI, Bathel Index; FSHD, facioscapulohumeral muscular dystrophy; QoL, quality of life.

All MD groups reported significantly lower scores than controls across quality of life, perceived functional ability and pain assessments, except in activities of daily living, where no differences were observed. In multiple group comparisons, QoL was 46–61 % lower in all MD groups compared with controls (*P* < 0·05, Figure 5(c)), pain was 64–77 % higher in all MD groups (*P* < 0·05, Figure 5(b)) and the LEFS was 67–80 % lower in all MD group (*P* < 0·05, Figure 5(e)). No differences were observed for Nutritional Knowledge Questionnaire or Short Food Literacy Questionnaire between groups or across multiple groups, data not presented.

### HBA1c and 25-hydroxyvitamin D

No significant differences in HbA1c or 25-hydroxyvitamin D levels were observed between MD and Control groups or across multiple subgroups, irrespective of supplementation status (online Supplementary Tables 1 and 2).

### Correlations between dietary intake, muscle strength and perceived function

**Figure 6.**
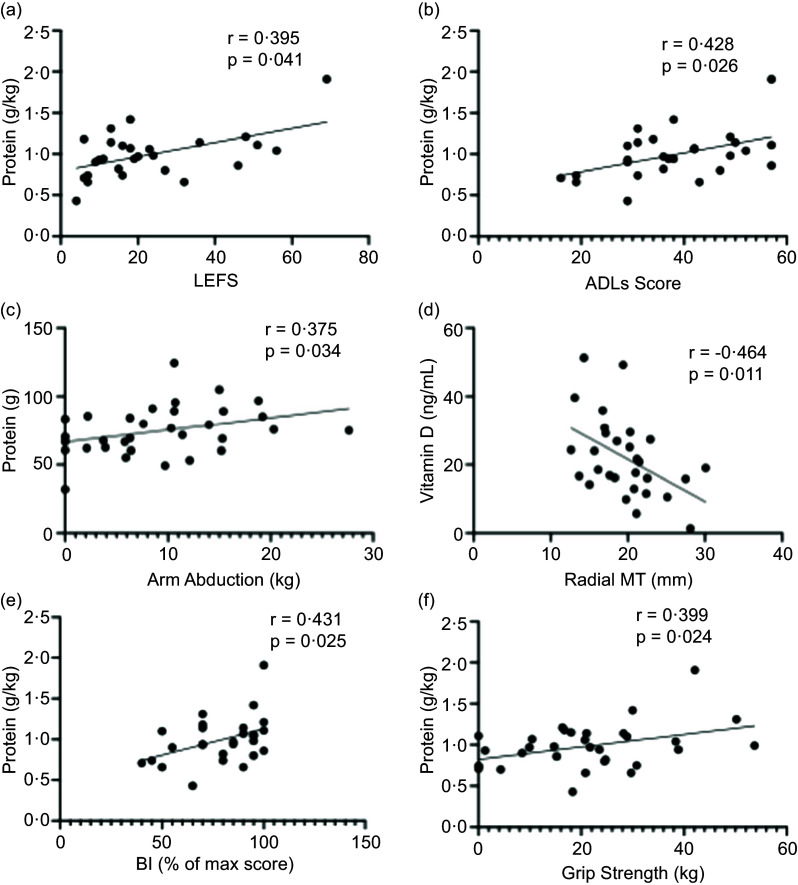
Correlations in MD illustrating the relationships between protein intake (g/kg) and (a) Lower Extremity Functional Scale (LEFS), (b) Activities of daily living (ADL), (c) Barthel Index (BI), (f) grip strength and (e) arm abduction. Additionally, (d) shows the correlation between serum 25-hydroxyvitamin D [25(OH)D] levels and radial muscle thickness. Pearson’s correlation coefficients (*r*) and corresponding *P* values are reported for each association. MD, muscular dystrophy; MT, muscle thickness.

**Figure 7.**
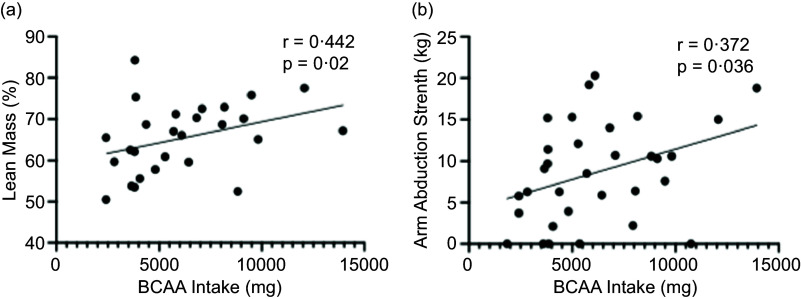
Correlations in MD illustrating the relationship between BCAA Intake (mg) and lean mass % (a) and AA strength (b) Pearson’s correlation coefficients (*r*) and corresponding *P* values are reported for each association. AA, shoulder abduction; BCAA, branched-chain amino acids; MD, muscular dystrophy.

Analysis of the correlations between macronutrient intake parameters and muscle strength measures revealed that absolute protein intake, positively correlated with both AA (*r* = 0·375, *P* = 0·034) and handgrip (*r* = 0·399, *P* = 0·026) strength in individuals with MD (Figure 6(c), (f)). Relative protein intake (g/kg) and protein as a percentage of RDI also showed positive correlations with AA and handgrip strength (data not shown). Furthermore, all protein intake parameters were positively associated with perceived functional measures, including the Bathel Index, ADL and the LEFS (Figure 6(a), (b), (e)). Branched-chain amino acids intake was moderately, positively correlated with both lean mass (%) (*r* = 0·442, *P* = 0·02, Figure 7(a)) and AA (*r* = 0·372, *P* = 0·036, Figure 7(b)), these correlations were not observed in the Control group. In the Control group, protein intake parameters correlated with Lower Extremity Functional Scale, ADL, pain Visual Analogue Scale and AA but not handgrip (data not shown).

### Correlations between HBA1c, 25-hydroxyvitamin D and muscle characteristics

Analysis of correlations between serum vitamin D and muscle characteristics revealed a negative correlation between serum vitamin D levels and radial muscle thickness in individuals with MD (*r* = –0·464, *P* = 0·011) (Figure 6(d)). This association was not observed in the Control group. However, in controls, serum vitamin D levels positively correlated with elbow flexion (*r* = 0·753, *P* = 0·001) and AA (*r* = 0·586, *P* = 0·022). No associations were seen between HBA1c and any other outcome measures in any group.

## Discussion

This study compared dietary intake between adults with MD and a healthy control group as well as probing associations between dietary habits and quality of life, physical function and muscle size. We show adults with MD have a greater energy intake, in relation to age-/sex-specific RDI and lower protein intake when compared with controls. Moreover, in MD adults, protein intake and branched-chain amino acids intake were positively associated with assessments of strength, perceived function and body composition, suggesting interventions increasing intakes of these nutrients may be beneficial. Widespread inadequate nutrient intakes in both MD and Control reflect the broader issue of poor diet quality in the UK^(85,86)^, suggesting aspects of poor nutritional practice are not MD specific.

Progressive atrophy in MD is attributed to an imbalance in protein metabolism, causing net negative protein balance^(87–89)^. Dietary protein modulates this by stimulating muscle protein synthesis and attenuating breakdown via insulinogenic effects, making it a potentially modifiable factor in MD management. Herein, adults with MD consumed 20 % less protein (relative to RDI) than controls, with 23 % failing to meet the UK’s RDI of 0·75 g·kg^−1^·d^−1^, more than double the inadequacy rate in controls (11 %). These findings align with prior reports indicating inadequate dietary protein in 5–53 % of dystrophic populations^(1,21,90)^. This suboptimal protein intake, within a group already exhibiting compromised skeletal muscle metabolism, may exacerbate catabolism and accelerate atrophy^(7,91)^. Notably, the UK’s protein recommendation may not adequately address the altered metabolic, immune and frailty-related profiles seen in MD. Higher protein intakes are associated with better health outcomes in older adults, including preserved lean mass and functional performance^(92)^. The ESPEN group recommends 1·2–1·5 g·kg^−1^ of protein daily for older or chronically ill individuals due to anabolic resistance and increased catabolic burden^(93)^. Similar factors are likely evident in MD, suggesting similar heightened requirements for protein. Adopting the lower ESPEN threshold (1·2 g·kg^−1^) would classify 85 % of our MD cohort as protein insufficient, underscoring a substantial nutritional gap. Importantly, however, our data showed moderate positive correlations between relative protein intake and strength/function outcomes in our MD cohort. Specifically, higher protein intakes were associated with better scores in perceived function (Bathel Index, ADL and LEFS) and greater grip and arm abduction strength (*r* = 0·38–0·43, Figure 7). Additionally, branched-chain amino acids intake was positively associated with lean mass percentage (*r* = 0·44), consistent with the anabolic role of BCAA, particularly leucine, in skeletal muscle protein metabolism; supplementation with leucine or leucine-rich proteins has been shown to promote muscle accretion or attenuate protein loss in muscle wasting conditions^(94)^. Although not indicative of a direct causal relationship, these findings suggest that elevated protein intake may support muscle strength and function in MD. In older adults, increased protein intake within ESPEN guidelines has been linked to preserved physical performance and reduced functional decline^(95)^, supporting the plausibility of similar effects in MD. Collectively, these findings indicate the majority of adults with MD consume insufficient dietary protein while elevated protein consumption is associated with greater muscle strength and perceived function. Future longitudinal trials are needed to evaluate whether targeted protein supplementation can offer viable interventions to mitigate muscle loss and strength/function decline in adults with MD.

Our findings also highlight MD-specific disparities in energy and macronutrient intakes. Relative to age- and sex-specific RDI, total energy intake was significantly higher in the MD group, with 89 % exceeding estimated energy requirements, compared with 35 % in controls. This contrasts with previous reports documenting hypophagia in MD populations^(1,21)^, which may be attributed to differences in cohort age affecting RDI classification. Earlier studies have linked reduced energy intake in MD to factors such as oropharyngeal dysfunction, upper limb weakness, gastrointestinal dysmotility and fatigue^(1,11)^, factors still present in our cohort. However, environmental influences, particularly high consumption of ultra-processed (UP) foods in the UK, may have modulated intake patterns. Prior research demonstrating reduced energy intake was conducted ∼20 years ago or in countries with lower UP food intake, such as France^(21)^. UP food consumption is associated with elevated total energy intake and obesity risk^(96,97)^: although mechanisms remain incompletely understood^(98)^. These foods are typically high in saturated fats and energy density^(99)^. In our cohort, UP and processed foods accounted for ∼43–44 % of total intake. Additionally, 82 % of MD participants exceeded fat RDI, with saturated fats comprising 37 % of total energy, well above current dietary guidelines, underscoring concerns regarding diet quality in MD.

Overweight and obesity are prevalent in MD^(100)^, with reduced physical activity alongside progressive muscle weakness recognised as primary contributing factors^(101)^. In our cohort, 31 % of MD were classified as overweight (BMI 25–29·9 kg/m^2^) and 28 % as obese (BMI ≥ 30 kg/m^2^), reflecting the impact of these dietary patterns. However, this prevalence of overweight and obesity (61 %) is comparable to the general UK population (64 %)^(102)^, suggesting MD may not confer a markedly different risk. This comparable rate may, however, be attributable to matched physical activity levels between the MD and control groups. Nevertheless, high relative energy and saturated fat in MD individuals should be considered in future interventions given their known contribution to metabolic complications including hypertension and type II diabetes.

In our cohort, 32 % of MD participants exhibited HbA1c concentrations within prediabetic or diabetic ranges (prediabetic 42–47 mmol/mol and diabetic > 47 mmol/mol), indicating impaired glycaemic status. Reduced skeletal muscle mass and quality are known to impair muscle glucose uptake and may therefore contribute to elevated HbA1c concentrations observed in the MD group. The high proportion of individuals at risk of type 2 diabetes in this cohort is of clinical concern, particularly given that type 2 diabetes accelerates age-related muscle loss in older adults^(103)^, while chronic hyperglycaemia exacerbates oxidative stress, both of which are already heightened in MD^(104)^. Previous research in Duchenne MD and Becker MD has demonstrated 20–30 % greater glycaemic responses to oral glucose tolerance testing compared with age-matched controls^(105)^, suggesting that insulin resistance may be a pathophysiological feature of MD. Historical data further support these findings^(106,107)^. Future research should explore glycaemic control across additional MD subtypes, similar to the current study. Moreover, the application of continuous glucose monitoring technology could facilitate free-living temporal measures of glycaemic variability providing a nuanced understanding of glycaemic control in clinical populations. This is of relevance in MD, where both disease-specific and external metabolic stressors may converge to impair glucose homeostasis.

In contrast to saturated fats, PUFA, particularly *n*-3 fatty acids, are associated with improved insulin sensitivity and reduced cardiovascular risk. In this study, > 70 % of participants across both MD and control groups failed to meet *n*-3 PUFA RDI. While no associations were found with strength or functional measures in the current study, *n*-3 PUFA have demonstrated anabolic and anticatabolic effects in skeletal muscles, including attenuation of disuse-induced atrophy^(108)^ and elevated nutrient-stimulated muscle protein synthesis^(109)^. In paediatric Duchenne MD, *n*-3 PUFA supplementation has delayed lean mass loss and reduced insulin resistance^(110)^ suggesting potential benefits in adult MD populations. Nevertheless, long-term trials are needed to assess sustained efficacy^(111)^. Notably, greater effort to meet the RDI seems to be necessary, not only in adults with MD but in general UK populations.

Beyond macronutrient imbalances, most participants in both MD and control groups exhibited suboptimal vitamin intakes. Vitamin C inadequacy was particularly evident in the MD cohort, with 51 % failing to meet the RDI compared with 0 % in controls. Notably, insufficiencies were most pronounced in the LGMD subgroup (76 %), which may be attributable to their comparatively lower fruit and vegetable consumption. As a potent antioxidant, vitamin C scavenges reactive oxygen species and preserves cellular integrity, mechanisms critical in countering oxidative stress, which is elevated in dystrophinopathies and implicated in impaired physical performance^(112–114)^. Although there is some evidence of improved strength, in FSHD, with a multi-ingredient supplement containing vitamin C^(24)^, we did not observe associations between this micronutrient and muscle strength or function. However, increase in intake to meet the UK RDI should be advised across the entire UK population.

Vitamin D is a further micronutrient with known effects on musculoskeletal health^(115–120)^. In this study, all MD individuals not actively supplementing vitamin D were classed as insufficient, and the majority (75 %) of those supplementing with ≥ 1000 IU/d remained insufficient. However, deficiency prevalence did not differ between MD and control cohorts, indicating a wider population issue rather than MD-specific pathology^(121)^. Given the reduced physical activity often observed in MD and the physiological relevance of vitamin D, continued supplementation should be encouraged to support musculoskeletal and systemic health in this population.

This study has several limitations, notably the lack of age-matching between MD and control groups. Although initially age-matched (MD cohort: mean age = 46, *n* 104), participant drop-out subsequently disrupted age comparability. To address this, analyses were aligned with sex/age-specific UK dietary and physical activity guidelines, in line with current evidence^(21,23,30)^. The cohorts were also matched on key variables including BMI, physical activity levels, ADL and nutrition knowledge, factors seldom controlled for in prior MD research^(1,21,105)^. Additionally, age was not identified as a cofounding factor in any muscle outcome measures. Dietary intake was assessed using self-reported, weighed food records, a method prone to underreporting^(122)^. To mitigate this, participants completed two multi-day food diaries (∼8 weeks apart), incorporating both weekdays and weekends to better capture habitual intake^(42,123,124)^. Heterogeneity within the MD cohort, encompassing varied subtypes and functional capacities, was partially address through subtype comparisons, revealing functional and dietary differences. However, intra-subtype variability, particularly within LGMD, limits specificity of nutritional recommendations. Larger studies targeting more homogeneous MD subgroups are needed to refine dietary guidance; however, recruitment of sufficient cohort sizes could be difficult. The cohort comprised individuals receiving regular therapy who could complete surveys and had no other health complications, representing a highly engaged and well-supported group; findings should therefore be interpreted cautiously when generalising to a typical clinic-based MD population. Despite limitations, this study provides valuable insight into current dietary practices across a spectrum of dystrophinopathies.

### Conclusion

This study identified significant nutritional disparities in adults with MD, including excessive energy intake and inadequate protein consumption compared with controls. Micronutrient inadequacies (e.g. *n*-3 and vitamin D) were common across both groups, suggesting widespread poor diet quality. Protein intake in MD was positively associated with strength and perceived function, highlighting its clinical relevance. Additionally, high saturated fat intake and deficiencies in key micronutrients such as vitamin C were observed. These findings support the development of and need for targeted dietary strategies, potentially involving multi-nutrient interventions (e.g. protein, *n*-3 and vitamin D) to mitigate disease-related symptoms. Despite shared dietary challenges with the general population, MD-specific metabolic demands warrant tailored nutritional guidance. Future research should focus on refining evidence-based dietary recommendations to improve long-term outcomes in dystrophic populations.

## Supporting information

Leaver et al. supplementary material 1 Leaver et al. supplementary material

Leaver et al. supplementary material 2 Leaver et al. supplementary material

Leaver et al. supplementary material 3 Leaver et al. supplementary material

Leaver et al. supplementary material 4 Leaver et al. supplementary material

## Supplementary material

For supplementary material/s referred to in this article, please visit https://doi.org/10.1017/S0007114525106119
