# Supplementary material for: Nutritional status is linked to muscle strength and perceived function in adults with muscular dystrophy: evidence for targeted nutritional interventions
Source: Br J Nutr. 2025 Dec 30;135(8):812–25. doi: 10.1017/S0007114525106119 (PMC13315556; doi:10.1017/S0007114525106119)
Supplement: Leaver et al. supplementary material 1 — Leaver et al. supplementary material [file S0007114525106119sup001.pdf]

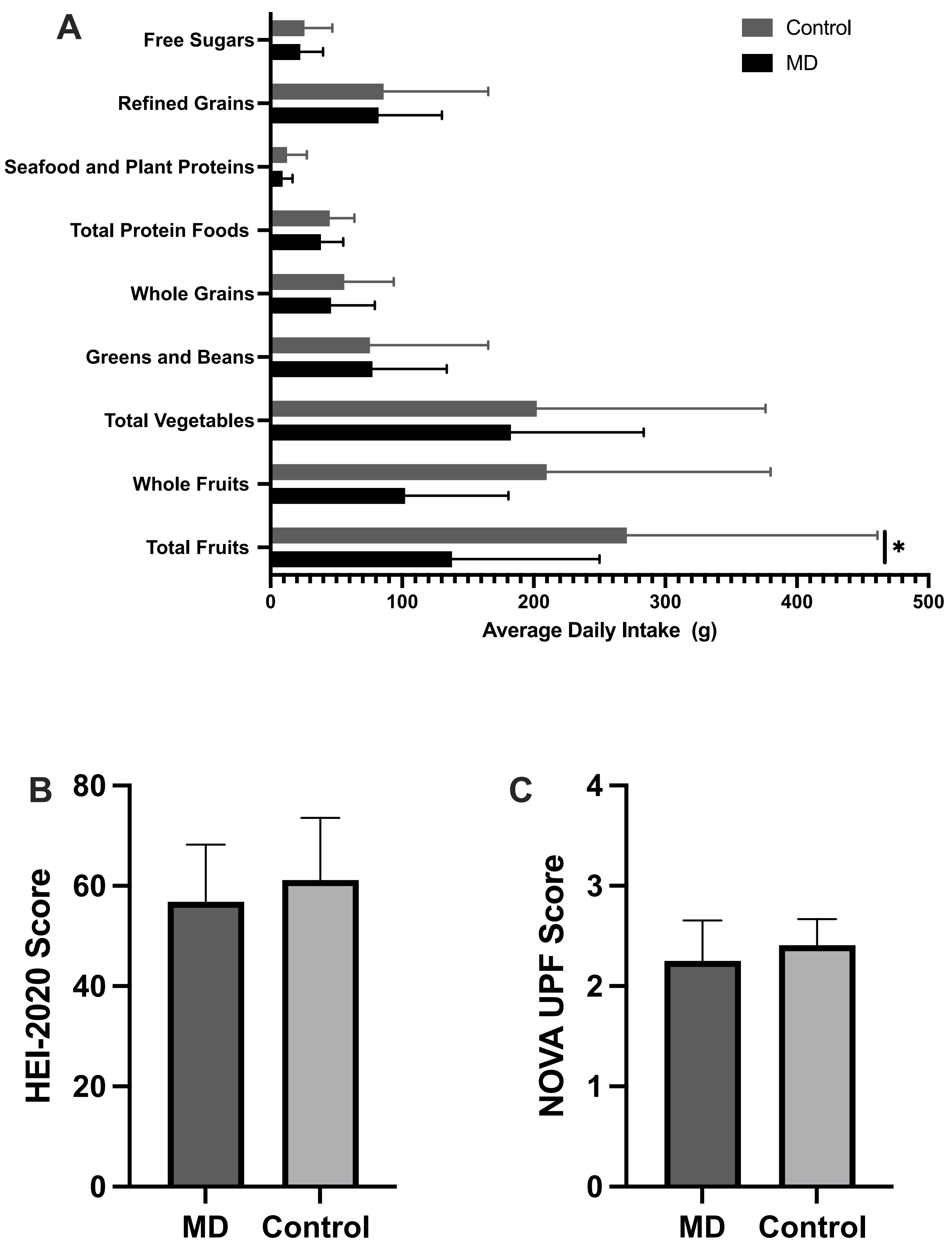

Supplementary Figure 1. Diet quality and ultra-processed food intake analysis.
Panel (A) presents individual food group components contributing to the Healthy Eating Index–2020 (HEI-2020). Panel (B) shows total HEI-2020 scores, reflecting overall diet quality. Panel (C) depicts NOVA classification scores for ultra-processed food (UPF) consumption. Data are presented as mean ± SD. MD significantly different from Control ($p < 0.05$).
