# Supplementary material for: Nutritional status is linked to muscle strength and perceived function in adults with muscular dystrophy: evidence for targeted nutritional interventions
Source: Br J Nutr. 2025 Dec 30;135(8):812–25. doi: 10.1017/S0007114525106119 (PMC13315556; doi:10.1017/S0007114525106119)
Supplement: Leaver et al. supplementary material 3 — Leaver et al. supplementary material [file S0007114525106119sup003.docx]

**Table 6.** 25(OH)D concentrations in MD and Controls groups, categorised by whether they use vitamin D supplements or not. Values are reported as mean serum 25(OH)D concentrations ± SD.

|  | **MD^1^ (supp^2^)** | **MD^1^ (no supp^3^)** | **Control (supp^2^)** | **Control (no supp^3^)** |
| --- | --- | --- | --- | --- |
|  | *(n=16)* | *(n=23)* | *(n=3)* | *(n=14)* |
|  |  |  |  |  |
| **Serum 25(OH)D (ng/mL)** | 28.13 ± 8.1 | 12.34 ± 5.87 | 29.07 ± 7.34 | 17.58 ± 10.45 |

^1^ MD, muscular dystrophy participants; ^2^ supp, supplement group, ^3^ no supp, no supplement group

**Table 7.** Vitamin D status of participants in the MD and Control groups, categorised by whether they use vitamin D supplements or not. Values are reported as mean serum 25(OH)D concentrations ± SD.

|  | **MD^1^ (supp^2^)** | **MD^1^ (no supp^3^)** | **Control (supp^2^)** | | **Control (no supp^3^)** | |
| --- | --- | --- | --- | --- | --- | --- |
|  | *(n=16)* | *(n=23)* | *(n=3)* | | | *(n=14)* |
|  |  |  | |  | |  |
| **Severe Deficiency *(<12ng/mL)*** | 1 | 11 | | 0 | | 6 |
| **Deficiency**  ***(12-20ng/mL)*** | 3 | 10 | | 0 | | 5 |
| **Insufficiency**  ***(20-30ng/mL)*** | 8 | 3 | | 2 | | 2 |
| **Sufficiency**  ***(30-50ng/mL)*** | 4 | 0 | | 1 | | 1 |
| **Possible Toxicity *(>100ng/mL)*** | 0 | 0 | | 0 | | 0 |

^1^ MD, muscular dystrophy participants; ^2^ supp, supplement group, ^3^ no supp, no supplement group
