# Supplementary material for: Nutritional status is linked to muscle strength and perceived function in adults with muscular dystrophy: evidence for targeted nutritional interventions
Source: Br J Nutr. 2025 Dec 30;135(8):812–25. doi: 10.1017/S0007114525106119 (PMC13315556; doi:10.1017/S0007114525106119)
Supplement: Leaver et al. supplementary material 4 — Leaver et al. supplementary material [file S0007114525106119sup004.docx]

**Table 8.** Glycated haemoglobin concentrations in MD and Control groups. Values are reported as mean glycated haemoglobin levels (HBA1c) ± SD.

|  | **MD^1^** | **Control** |
| --- | --- | --- |
|  | *(n=32)* | *(n=17)* |
| **HBA1c^2^ (mmol/mol)** | 40.68 ± 10.13 | 35.38 ± 6.44 |
|  | **MD^1^** | **Control** |
|  | *(n=32)* | *(n=17)* |
| **Normal (<42 mmol/mol)** | 21 | 16 |
| **Prediabetic (42-47 mmol/mol)** | 3 | 0 |
| **Diabetic (>47 mmol/mol)** | 8 | 1 |

^1^ MD, muscular dystrophy participants; ^2^ HBA1c, glycated haemoglobin
